# IMPACT OF PROJECT ECHO ON SELF-ASSESSED KNOWLEDGE IN LONG-TERM CARE AND PRIMARY CARE WORKFORCES

**DOI:** 10.1093/geroni/igae098.4217

**Published:** 2024-12-31

**Authors:** Jacqueline Telonidis, Cherie Brunker, Christopher Hernandez, Kara Dassel, Linda S Edelman

**Affiliations:** University of Utah, Salt Lake City, Utah, United States; Internal Medicine Division of Geriatrics, Salt Lake City, Utah, United States; University of Utah, Salt Lake City, Utah, United States; University of Utah, Salt Lake Cty, Utah, United States; College of Nursing, Salt Lake City, Utah, United States

## Abstract

Project ECHO is a widely accepted method for transforming care through education. The Utah Geriatric Education Consortium (UGEC), a Health Resources and Services Administration funded Geriatric Workforce Enhancement Program, developed a monthly Age-Friendly ECHO series to educated clinicians about the Institute for Healthcare Improvement Age-Friendly Health Systems Initiative 4M’s Framework. The purpose of this program evaluation was to measure knowledge gained over the 12-month series ending in June 2024.

**Methods:**

We used retrospective pre-post testing via Zoom polling to measure knowledge gained for each ECHO topic. This approach measures participants’ learning after the session by asking them to assess their knowledge from two viewpoints – BEFORE and AFTER participating.

**Results:**

The UGEC hosted 11 ECHOs with a total of 461 participants: Allied Health (3.04%), Behavioral Health (20.61%), Medicine (10.63%), Nursing (20.17%), Paraprofessionals (6.07%), and other professions including facility administrators, informal caregivers, etc. (39.48%); students made up 3.08% across all disciplines. Overall, results revealed significant improvements in knowledge gained (t = -12.5273, df = 208, p < 0.001). The greatest changes were observed for presentations about Advanced Treatment Options for Parkinson’s Disease (t = -5.9847, df = 18, p < 0.001), Helping Patients Age Well (t = -5.4349, df = 20, p < 0.001), and Keys to Addressing Ageism (t = -4.8190, df = 18, p < 0.001). Conclusions: Retrospective pre-post is a valid method to measure learning compared to a traditional pre-post approach. Project ECHO is an effective learning model for best-practice dissemination to increase knowledge about the 4Ms Framework.

